# Discussion on Dr. Cunningham’s Paper

**Published:** 1894-01

**Authors:** G. W. Christian

**Affiliations:** Burnet


### DISCUSSION OF DR. CUNNINGHAM'S PAPER, BY DR. G. W. CHRISTIAN, OF BURNET.

Dr. Paine has so thoroughly covered the subject, that but little remains to be said. I wish to commend the paper, and to endorse what Dr. Paine has said, most especially as to the choice of cathartics. We not only want the bowels open, but we want to supplement kidney action, for the relief of the hydræmic condition which is a part of the pathology of those cases. And as to the production of premature labor, I can't comprehend any opposition to such practice, from a well informed and sensible physician, when he is met by a failure after a careful and intelligent employment of the course suggested by Dr. Paine. I like the doctor's suggestion, as to the use of hot air, the skin is our most valuable auxiliary in these cases, and we all appreciate the value of heat as a stimulant to its action. I have been in the habit of using in such cases, a sheet wet in hot water, with hot rocks, etc., but we appreciate the superior merits of hot air, and the ingenious method of Dr. Paine is easy of application, and generally accessible, even to the back-woods practitioner. While we would differ with Dr. Cunningham in some respects, in the treatment of his cases, the doctor's success has been remarkably good, and demonstrates the fact of intelligent management. Dr. Paine, in speaking of the treatment of albuminuria, extols iron, but I think the doctor would use his iron to lessen the albuminuria previous to the convulsive attack. When the seizure has come, doctor, you would not give iron, would you? Paine answers "no." But direct your efforts towards stimulating the bowels and skin. Paine, "yes." I have in mind a case, that illustrates the importance of physicians not only being familiar with the remedies for a condition, but being able to discriminate as to the time, for the application of such remedies. Not long since, I saw in a neighboring city, a beautiful and accomplished young wife, of a proud and noble husband, who after years of loving courtship were happy in the contemplation of the fruits of their union, with all hopes suddenly blasted by the dread disease under discussion, the mother after a few hours of apparent normal labor, went off into a deep uremic coma, from which she never aroused. A few hours later her attendants delivered her of a fine living child, but practically nothing had been done that could have had much influence in relieving the mother of the condition which had prostrated her. She was, and had been dosed with calomel and iron, with some other drugs which I do not now remember, all of which were good in their place, but too inert and slow in action, to meet the demands of a storm like this; eight or ten hours of valuable time had been frittered away and no action of bowels or skin had been secured. A few hours more closed the struggle, a motherless babe, a heart broken husband. None can say that she could have been saved, but you all will unite with me in saying, "what a pity she did not have the benefit of a quick and active purge and a good snbstantial sweat." Thanking you, gentlemen, for your patient hearing, I leave the subject, hoping to hear from others.

[The paper was discussed also by Prof. J. F. Y. Paine, M. D., Galveston; Dr. C. O. Weller, Austin, and other members, and we were promised a synopsis of their remarks, but to date Dr. Christain's is the only report we have received.—Ed.]

